# Watson’s Lectures on the Practice of Physic

**Published:** 1845-11

**Authors:** 


### WATSON'S LECTURES ON THE PRACTICE OF PHYSIC.

It is only a little more than a year since we had the pleasure of calling the attention of our readers to the former edition of this excellent system of the Principles and Practice of Physic. The fact that a second edition has been so soon called for, is the best proof that the profession has ratified the favorable opinion of the medical press concerning the work. Dr. Condie has made valuable additions to it, adapting it more fully to the wants of the American profession. Some things he has omitted which we could have wished had been incorporated with these valuable lectures: we shall expect to find them in a new edition which, doubtless, will soon be called for. Y.

